# Dynamic Changes in Genome-Wide Histone3 Lysine27 Trimethylation and Gene Expression of Soybean Roots in Response to Salt Stress

**DOI:** 10.3389/fpls.2019.01031

**Published:** 2019-09-10

**Authors:** Lei Sun, Guangshu Song, Weijun Guo, Weixuan Wang, Hongkun Zhao, Tingting Gao, Qingxue Lv, Xue Yang, Fan Xu, Yingshan Dong, Li Pu

**Affiliations:** ^1^College of Agriculture, Northeast Agricultural University, Harbin, China; ^2^Soybean Research Institute, Jilin Academy of Agricultural Sciences, Changchun, China; ^3^Maize Research Institute, Jilin Academy of Agricultural Sciences, Gongzhuling, China; ^4^Biotechnology Research Institute, Chinese Academy of Agricultural Sciences, Beijing, China

**Keywords:** salt stress, RNA-seq, ChIP-seq, histone methylation, histone modifiers, soybean

## Abstract

Soybean is an important economic crop for human diet, animal feeds and biodiesel due to high protein and oil content. Its productivity is significantly hampered by salt stress, which impairs plant growth and development by affecting gene expression, in part, through epigenetic modification of chromatin status. However, little is known about epigenetic regulation of stress response in soybean roots. Here, we used RNA-seq and ChIP-seq technologies to study the dynamics of genome-wide transcription and histone methylation patterns in soybean roots under salt stress. Eight thousand seven hundred ninety eight soybean genes changed their expression under salt stress treatment. Whole-genome ChIP-seq study of an epigenetic repressive mark, histone H3 lysine 27 trimethylation (H3K27me3), revealed the changes in H3K27me3 deposition during the response to salt stress. Unexpectedly, we found that most of the inactivation of genes under salt stress is strongly correlated with the *de novo* establishment of H3K27me3 in various parts of the promoter or coding regions where there is no H3K27me3 in control plants. In addition, the soybean histone modifiers were identified which may contribute to *de novo* histone methylation and gene silencing under salt stress. Thus, dynamic chromatin regulation, switch between active and inactive modes, occur at target loci in order to respond to salt stress in soybean. Our analysis demonstrates histone methylation modifications are correlated with the activation or inactivation of salt-inducible genes in soybean roots.

## Introduction

Environmental changes affect the organisms in a wide range of situations (Lopez-Maury et al., 2008). Among the abiotic stress factors, salt stress is a well-known factor restricting germination and growth, seriously threatens the productivity of crops. Soybean, *Glycine max*, is one of the most important crops with source of protein and oil in the human and animal diet, however its productivity is significantly affected by field condition such as soil salinity (Phang et al., 2008). In the northeast China, soybean used to be a major crop, and breeding soybean for tolerance to high sodic conditions is important in some regions of China and the world. Therefore, understanding the molecular mechanism of the soybean tolerance to salt stress has been a major topic for crop scientists (Zhang et al., 2013).

Plants respond to abiotic stress by activation or inactivation of specific sets of genes to induce certain molecular signaling pathways which rapidly alter physiological reactions and expression initiation of responsive genes. Gene expression is directly influenced through chromatin states, which is closely associated with epigenetic regulation including histone variants, histone post-translational modifications, and DNA methylation (Schwartz et al., 2010; Henikoff and Shilatifard, 2011; Lauria and Rossi, 2011). The modifications of the histone amino-terminal tails are involved in assisting nucleosome remodeling as well as recruitment of specific transcription factors. Specific amino acids within the N-terminal regions of histones are targets for a number of covalent modifications, including methylation, phosphorylation, ubiquitination and acetylation. Some of these marks, for example, acetylation of lysine 14 of histone H3 (H3K14ac) or trimethylation of lysine 4 of Histone3 (H3K4me3), are generally associated with open, actively transcribed genomic regions, whereas others, such as H3K9me3 or H3K27me3, are indicative of a repressed chromatin state (Zhang et al., 2007; Li et al., 2008; Charron et al., 2009; Zhang et al., 2009; He et al., 2011).

The epigenetic changes including DNA methylation and/or histone modifications are associated with altered gene expression for defense responses under abiotic (e.g., salt) stress (Alexandre et al., 2009; Chinnusamy and Zhu, 2009; Ding et al., 2009; Zong et al., 2013; Chen et al., 2014). In plants, there are increasing studies of regulating gene expression by histone modification under various stresses (Chinnusamy and Zhu, 2009; Kumar and Wigge, 2010; Luo et al., 2012a; Feng et al., 2016; Deng et al., 2017). In crop breeding, it is hard to keep balance of disease resistance and yield. Recent studies showed that the rice *Pigm* locus contains a subset of genes encoding nucleotide-binding leucine-rich repeat (NLR) receptors. These receptors can lead to durable resistance to the fungus without productivity penalty through DNA methylation regulation (Deng et al., 2017). To cope with environmental stresses, plants often adopt a memory response when facing primary stress for a quicker and stronger reaction to recurring stresses. Feng et al. found that salt stress-induced proline accumulation is memorable. HY5- dependent light signaling through H3K4me3 modification on a Δ1-pyrroline-*5-carboxylate synthetase 1* (*P5CS1*) is required for such a memory response (Feng et al., 2016).

The covalent modifications were deposited or erased from target loci by the histone modifiers including histone methyltransferase (HMTs) and histone demethylases (HDMs). All the known HMTs in plants have a highly conserved domain, SET (Su(var)3-9, Enhancer-of-zeste, Trithorax) which was also named as SDG (SET domain groups) proteins (Ng et al., 2007; Thorstensen et al., 2011). Many epigenetic modifiers’ function has been well characterized. It has been reported that some modifiers have been shown to be integrated in abiotic stress signaling pathways (Schubert et al., 2006; Grini et al., 2009; Jeong et al., 2009; Guo et al., 2010; Ding et al., 2011; Lu et al., 2011; He et al., 2012; Yao et al., 2013; Gu et al., 2014; Cui et al., 2016). A plant trxG factor, *Arabidopsis* homolog of trithorax1 (ATX1) with H3K4me3 methyltransferase activity can promote transcription initiation by recruiting RNA Polymerase II (Alvarez-Venegas and Avramova, 2005; Saleh et al., 2008). ATX1 was found to be involved in drought stress signaling in both ABA dependent and ABA-independent pathways, and an *atx1* mutant was shown to be hyposensitive to drought stress (Ding et al., 2009; Ding et al., 2011). Therefore, chromatin modifications and epigenetics are directly linked to plants’ responses to environmental cues.

It is important to note, however, that most of the current studies focus on epigenetic modifications at individual stress genes in plants. Second, there are more and more studies on *Arabidopsis*, rice, and maize, but limited knowledge of regulation of salt stress response through chromatin modifications in soybean plants. Moreover, there are no data on genome-wide modification patterns in regard to response to stress in soybean plants. In this study, we provide a global view of H3K27me3 patterns in chromatin isolated from soybean roots with or without salt stress treatment. Genome-wide expression patterns in control and salt stressed soybean were compared with changes in the H3K27me3 levels of nucleosomes on stress-induced differentially expressed genes. Using chromatin immunoprecipitation (ChIP) of H3K27methylation antibodies combined with genome-wide sequencing (ChIP-seq), we revealed different dynamic changes in H3K27me3 profiles taking place upon salt stress. The specific patterns of the H3K27me3 distributions including *de novo* methylation at up-regulated and down-regulated genes were identified during the stress treatment. Moreover, we provide a comprehensive overview of the histone modifiers which may work together to regulate differential H3K27me3 modification leading to activation or inactivation of gene expression during salt stress in soybean.

## Materials and Methods

### Plant Materials and Growth Condition

The *Glycine* line, *Glycine max* Williams 82, was used in this study. Seeds were sterilized with 75% ethanol and then germinated in pots filled with coconut fiber. Soybean seedlings were grown in soil in an incubator with 25/20°C (light/dark) and 16/8h (light/dark) cycles until the second trifoliate leaves started expand. For the salt stress treatment, the uniformly growing plants were kept in 0, 50, 75, 100, 150, and 200 mM/L of NaCl solutions for 30 h. After the treatment, the root tissues were harvested and frozen in liquid nitrogen. As a control, the untreated seedlings (0 mM/L) were planted and harvested at the same time with the stress-treated plants. The 100 mM/L salt treated seedlings were used for RNA-seq and ChIP-seq analysis since the phenotypic differences were clear at this concentration which is also commonly used for salinity test on soybean (Belamkar et al., 2014; Zeng et al., 2019). Three replicates of the root samples both from control and 100 mM/L salt treatment were prepared for consistency of the analysis.

### RNA-seq Library Construction and Analysis

Total RNA was extracted from the root of soybeans with TRIzol reagent (Invitrogen) according to the manufacturer’s instructions. Library making, RNA-seq and data analysis were performed as described previously (Xu et al., 2018). PolyA^+^ libraries were constructed using Illumina’s TruSeq Stranded mRNA Library Prep Kit. The size and quality of the resulting libraries were examined using a Bioanalyzer 2100 and cDNA libraries from the RNA samples were prepared for high throughput Illumina sequencing. Paired-end reads were generated with the Illumina HiSeq 2500 system. Three independent biological sample replicates were employed. The RNA sequencing reads were aligned to the *Glycine max* reference genome (*Glycine* max Wm82.a2.v1) using TopHat2 (Kim et al., 2013). Genes that met the criterion of a detectable expression signal in control or salt plants were further analyzed. The fold change (FC) was calculated by comparing the expression level of the salt samples to control (salt/control). Briefly, the “|Log2FC| > 1 and p-adj < 0.05” was used as the threshold to judge the significance of gene expression difference. Genes that display a greater than 2-FC in the salt-treated were designated as up- or down-regulated if the salt RNA level was higher or lower than that of control plants, respectively.

### Real-Time Quantitative RT-PCR (qPCR)

RT-qPCR was performed as described previously with minor modifications (Xu et al., 2018). cDNAs were reverse transcribed with oligo (dT) from the total RNAs. RT-qPCR reaction was carried out in a QuantStudio 6 Real-Time PCR system (Applied Biosystems). At least three independent experiments employing biological replicates were performed and three technical replicates were done for each sample. Amplification of *Tubulin* (*Tub*) was used as an internal control to normalize all data. Quantification was determined by applying the 2^−ΔCt^ formula (Pu et al., 2008). All gene-specific primers are listed in **Supplementary Table 1**.

### Chromatin Immunoprecipitation (Chip) Assays and ChIP-seq Analysis

ChIP assay was performed from approximately 2 g of soybean roots as previously described (Kim et al., 2012b; Xu et al., 2018). Briefly, fresh tissues of whole seedlings were infiltrated in 1% formaldehyde solution under a vacuum for 20 min to cross-link the chromatin. The reaction was stopped by adding 0.1 M glycine. Formaldehyde fixed tissues were ground in liquid nitrogen, nuclei isolated, chromatin extracted and sheared by sonication (Diagenode, Bioruptor Plus; 1 min on and 30 s off for 15 min) to generate 0.5 to 2 kb DNA fragments. The aliquot of 1–2 μl of mix DNA samples and electrophoretic was used to determine the sonication efficiency and average size of DNA fragments. A smear from 200–2,000 bp, but concentrated 500 bp was observed in the sonicated samples and for further analysis. Anti-H3K27me3 (Millipore, #07-449) antibody was used to immunoprecipitate the fragmented chromatin (IP, 200 μl of IP solution plus 1μl of antibody as 200 times dilution). Cross-linking of IP was reversed with 5 M NaCl, and DNA was precipitated with 100% EtOH. For the Input control (Input), 5M NaCl was added to 0.5% of total chromatin before immunoprecipitation to reverse the cross-linking and DNA was precipitated with 100% EtOH. The relative amount of DNA was determined using a spectrophotometer (NanoDrop, ND1000). ChIP purified DNA was amplified for 14 cycles using the Sigma Genomeplex Whole Genome Amplification (WGA2) kit following the manufacturer’s protocol (Sigma-Aldrich Co, Catalog Number WGA2). More than 20 ng of IP DNA from each sample was used for library generation following the manufacturer’s instructions. Three independent biological sample replicates were employed.

Library construction and deep sequencing were performed as described previously (Wang et al., 2016; Xu et al., 2018). ChIP DNA samples described above were prepared for high throughput Illumina sequencing (one hundred and fifty pair-end read sequencing). The ChIP-seq data was analyzed as described previously (Wang et al., 2016; Xu et al., 2018). The first 30 base pairs from the 5’ end containing primer or adapter sequences were trimmed. The 3’ end of the sequencing reads were trimmed based on base-call quality using the BWA quality trim algorithm (Li and Durbin, 2009). The sequencing reads were aligned Glycine max reference genome (Glycine max Wm82.a2.v1). Only uniquely mapped reads that mapped to one location of the genome only (as opposed to those that mapped to multiple reads) were retained for peak calling. Three biological replicates were performed for each sample. Each input was used as a control for peak calling for each sample using MACS 1.4 (Zhang et al., 2008). The statistical identification of peaks was performed for each sample using MACS with the default 10^−5^ *p*-value cutoff. The three replicates results were overlapped using BedTools (Quinlan and Hall, 2010). The resulting BED format files that contain the peak location were visualized with the Integrated Genome Viewer (Robinson et al., 2011; Thorvaldsdottir et al., 2013).

ChIP-seq results were verified by ChIP-qPCR for selected genes as previously described (Xu et al., 2018). The relative amounts of Input and IP DNA of all samples were determined using a spectrophotometer (NanoDrop, ONE C). The diluted ChIP DNA was analyzed by qPCR according to the procedure described above for RT-qPCR. Three replicates were done for each sample. Quantification was determined by applying the 2^−Ct^ formula (SuperArray ChIP-qPCR user manual; Bioscience Corporation). Average immunoprecipitates from chromatin isolated independently are expressed on graphs as percentage of corresponding input DNA, with error bars representing the standard deviations. All gene-specific primers are listed in **Supplementary Table 1**.

The *p*-value for the gene expression changes of methylated or *de novo* H3K27me3 genes in salt-treated soybean was calculated by using hypergeometric statistical test as described previously (Xu et al., 2018).

### Plasmid Constructions and *Arabidopsis* Transformation

The full length coding sequence of the *Glyma.17G022500* gene was amplified, and inserted into p*CAMBIA1301*, a binary vector, under control of the 35S promoter. The resulting vector was mobilized into *Agrobacterium tumefaciens* strain GV3101. Transformation of *Arabidopsis* wild-type Columbia plants was carried out by the floral dip method as described previously (Sanchez et al., 2009). Transgenic plants were first screened on MURASHIGE and SKOOG (MS) medium supplemented with 50 mg/L hygromycin. Seeds from each T1 plant were individually collected. Selected T2 plants were propagated, and homozygous overexpression lines were confirmed by genotyping analysis. T3 progeny homozygotes were obtained for further analysis.

## Results

### Gene Expression Change in Soybean in Response to Salt Treatment

**Figure 1:**
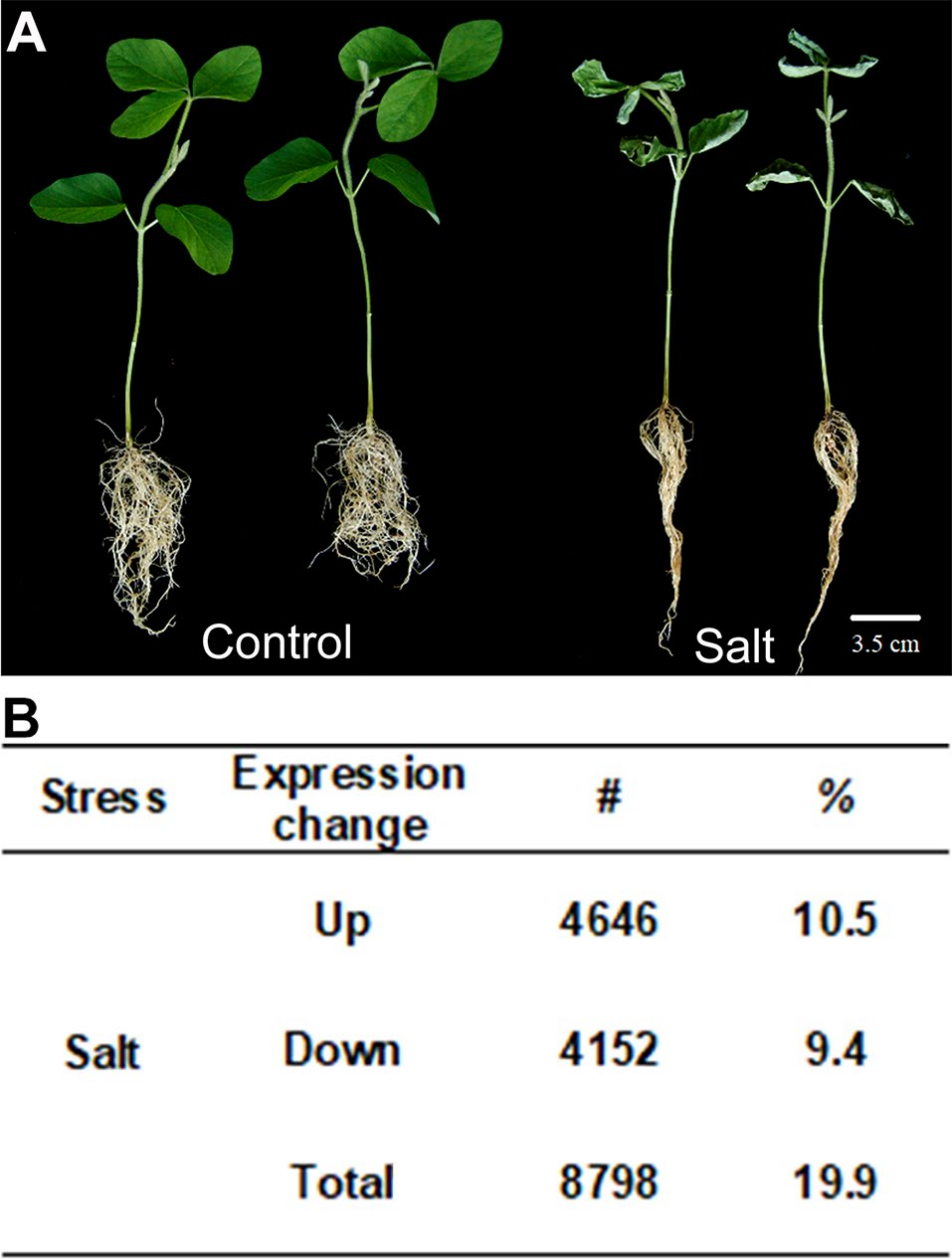
Soybean plants treated with salt stress and transcription profile analyzed by RNA-seq. **(A)** Salt treatment (100 mM of salt) on seedling of soybean (salt) and non-stressed control (control). **(B)** Gene expression changes in salt-treated soybean compared to control plants. Up means number and percentage (%) of genes up-regulated and Down means down-regulated relative to WT with *p*-value < 0.05. The total number of genes investigated is 44,346. Total indicates the total number of mis-regulated genes, i.e., total number of up- plus down-regulated genes. Bar = 2 cm.

Salt stress is a major abiotic stress that limits the yield of many crop species. In many plants, roots are the primary site of salinity perception. To better understand the mechanisms active in the response of roots to salt stress, we studied salt response in soybean with different concentrations of salt treatment (see Materials and methods section). We first evaluated the salt concentration that stressed soybean growth. Three biological replicates were subjected observed the phenotypes of salt-treated plants and found as the concentration of salt stress increased, root growth was increasingly retarded. As a result, we selected to grow roots in 100 mM salt to study the impact of salt stress on gene expression in soybean (**Figure 1A**). We employed RNA-seq technology to analyze genome-wide mRNA transcript levels in soybean roots under 0 mM (control) and 100 mM of salt treatment (salt). The RNA samples from the soybean roots grown with and without salt were sequenced by the Illumina Genome Analyzer. For each sample, we obtained approximately 42–54 million reads, of which 89.15–96.65% were mapped to the soybean reference genome (**Supplementary Table 2**).

From the sequence alignment data, the expression quantification for each sample was calculated using Cufflinks (Trapnell et al., 2013). To identify the salt responsive genes, a core set of differentially expressed genes (DEGs) under salt stress in soybean were examined. We classified them as up- or down-regulated genes with statistically significant two-fold expression changes in the samples treated with 100 mM salt compared to 0 mM control plants. A total of 44,346 soybean genes with confident expression were analyzed (**Supplementary Table 3**). Out of these genes, 8,798 (19.9%) were found to be differentially expressed genes under salt treatment compared to control plants, in which 4,646 genes are up-regulated and 4,152 were down-regulated (**Figure 1B**). There are a little bit more up-regulated genes than down-regulated genes in soybean roots after salt treatment.

### GO Analysis of Salt Response Gene in Soybean

**Figure 2:**
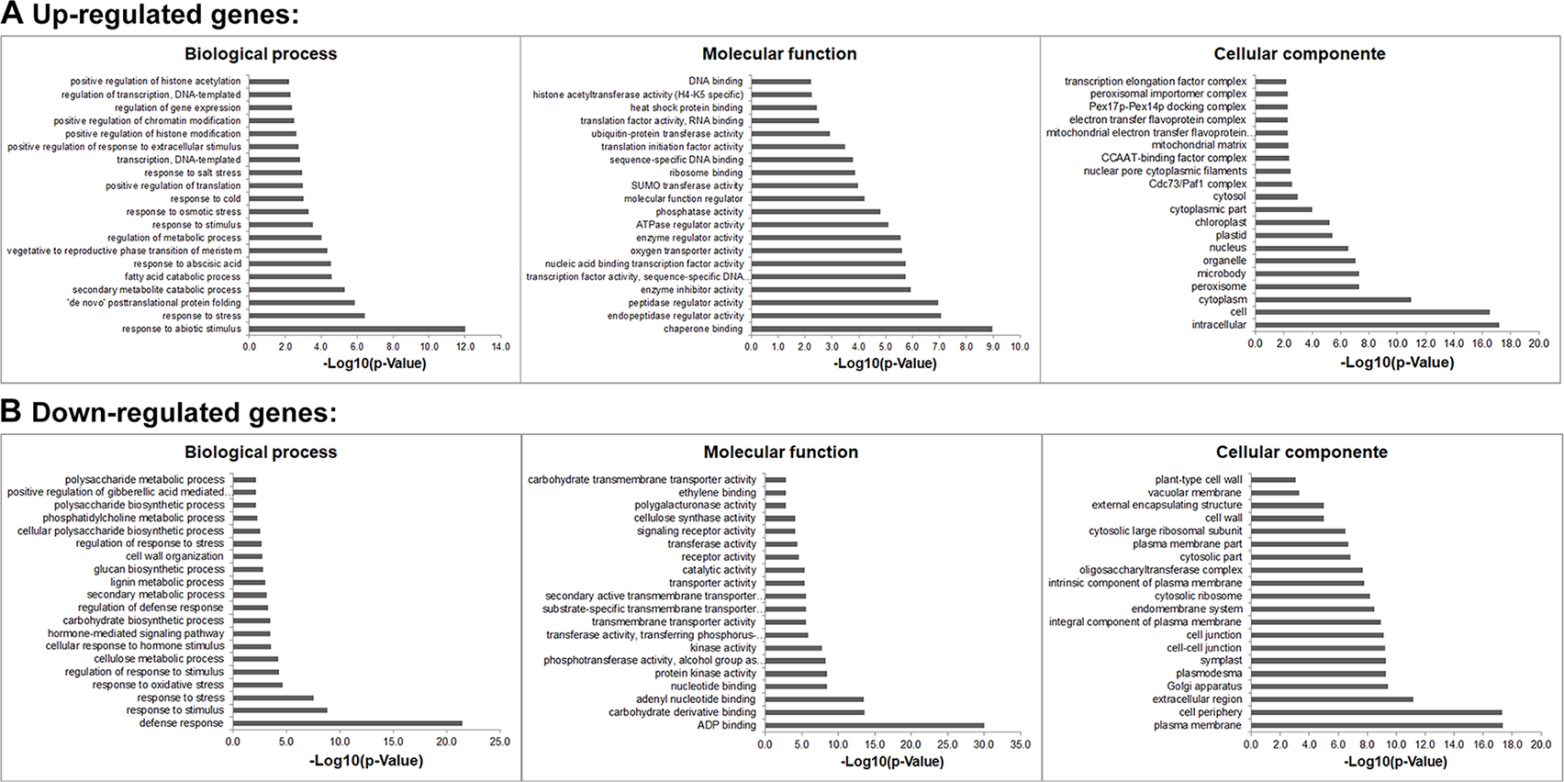
Gene Ontology (GO) study of up- and down-regulated genes under salt stress in soybean. The agriGO program (Tian et al., 2017) was used to identify significantly enriched molecular functions, biological processes and cellular component amongst the mis-regulated (up- or down-regulated) genes (*p*-value < 0.01). The terms were ranked by *p*-value.

**Figure 3:**
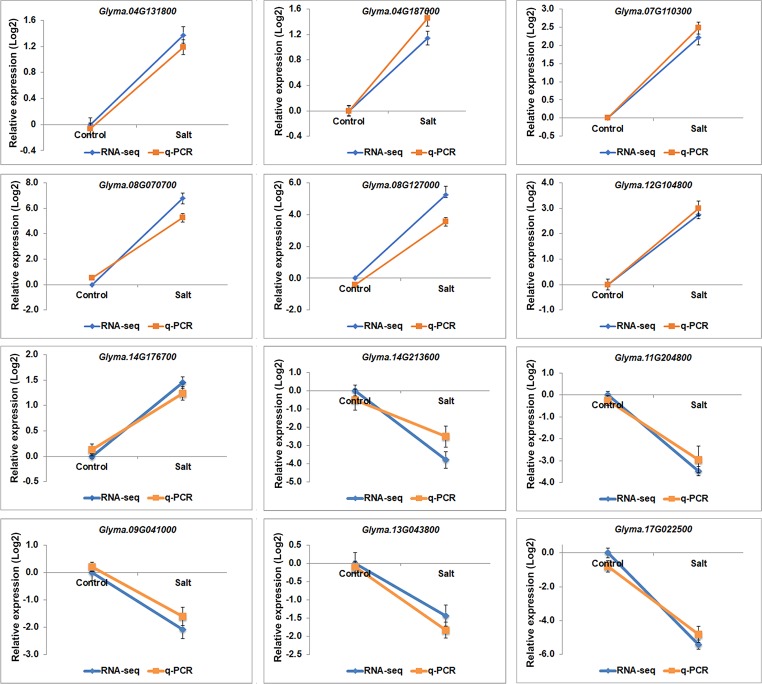
The gene expression profile of selected salt stress genes analyzed by RNA-seq and q-PCR. mRNA expression levels of 12 selected salt stress genes with differential expression levels, *Glyma.04G131800*, *Glyma.04G187000*, *Glyma.07G110300*, *Glyma.08G070700*, *Glyma.08G127000*, *Glyma.12G104800*, *Glyma.14G213600*, *Glyma.11G204800*, *Glyma.09G041000*, *Glyma.13G043800*, *Glyma.17g022500* and *Glyma.14G176700*, in control and salt-treated soybean. Graphs show the relative expression levels analyzed by RNA-seq and by qPCR which normalized to a *Tubulin* (*Glyma.05G203800*) reference gene. Error bars represent standard deviation (SD).

Gene ontology (GO) analyses showed that the DEGs under salt stresses occur in many functional groupings (**Figure 2**). The heat-map revealed different GO categories, such as transcriptional regulation, response to stress, defense response, regulation of defense response, and histone methylation represented by the up-regulated enriched genes in these categories (**Figure 2**). Compared to up-regulated genes, down-regulated genes were mainly enriched in metabolic processes. Notably, we found that except defense response, most of the GO categories of up- and down-regulated genes showed an opposite and comparable profile under salinity condition (**Figure 2**), which indicated that salt stress can cause differential and specific gene regulation in order to respond to threatening environmental factors. To explore the molecular mechanism underlying the salt response in soybean, we further analyzed mis-regulated genes whose functions are involved in salt response. Among mis-regulated genes, there were 93 genes which are closely related to salt stress response, in which 53 genes are up- and 40 are down-regulated respectively (**Supplementary Table 3**). To confirm the RNA-seq results, we examined the RNA levels of two known soybean genes, *Glyma.03G226000* and *Glyma.03G171600* (**Supplementary Figure 1**) and 11 selected salt|responsive genes, *Glyma.04G131800*, *Glyma.04G187000*, *Glyma.07G110300*, *Glyma.08G070700*, *Glyma.08G127000*, *Glyma.14G213600*, *Glyma.11G204800*, *Glyma.09G041000*, *Glyma.13G043800*, *Glyma.17g022500* and *Glyma.14G176700*, by RT-quantitative PCR (qPCR) (**Figure 3**). The expression levels determined by qPCR and those by RNA-seq analysis were highly correlated (**Figure 3**), indicating that the results obtained by the independent methods are consistent.

Transcription processes are carried out by transcription factors (TFs). To identify potential TFs involved in salt stress, we then analyzed expression patterns of genome-wide TFs. There are 3017 annotated transcription factors in soybean with expression data belonging to over 50 TF families such as homeodomain, zinc finger, WRKY, SET domain, MYB, MADS, AP2-EREBP, bHLH, NAC, bZIP and GRAS (**Table 1**) (Perez-Rodriguez et al., 2010; Jin et al., 2017). We found 513 TFs up-regulated and 491 are down-regulated under salt treatment, respectively (**Table 1**), which is consistent with the whole expression pattern of RNA-seq. Genes belonging to the bHLH, bZIP, ERF, GRAS, MYB, MYB-related, NAC, and WRKY family represent most of the differentially expressed TFs (**Table 1**). The bHLH, Ethylene Response Factor (ERF) and MYB represented the highest number of significantly expressed genes under salt treatment conditions. GO analyses showed 10 TFs investigated here belong to the category of salt stress response genes, in which 8/10 of genes are MYB TFs, such as *Glyma.12G104800*, *Glyma.16G073000*, *Glyma.01G107500*, *Glyma.15G236400*, *Glyma.06G097100*. This is consistent with previous studies that MYB TFs have been known to regulate salt stress response in plants (Yang et al., 2012; Cui et al., 2013; Li et al., 2016; Wei et al., 2017). Notably, the fold changes of some TFs were significantly higher than control plants (**Supplementary Table 3**). To verify the RNA-seq results, we examined the RNA level of one MYB gene, *Glyma.12G104800*, by qPCR analysis (**Figure 3**). The expression level determined by RT-qPCR and RNA-seq were highly consistent (**Figure 3**), confirming the results of the genome-wide analysis.

**Table 1:** Number of transcription factors under salt stress up- or down-regulated at least 2-fold in soybean.

Family	Total number	Up		Down	
		#	%	#	%
AP2	45	10	22.2	7	15.6
ARF	56	18	32.1	11	19.6
ARR-B	26	7	26.9	1	3.8
B3	42	6	14.3	4	9.5
BBR-BPC	10	2	20.0	0	0.0
BES1	15	6	40.0	1	6.7
bHLH	274	50	18.2	37	13.5
bZIP	140	20	14.3	20	14.3
C2H2	188	33	17.6	36	19.1
C3H	75	13	17.3	7	9.3
CAMTA	15	1	6.7	1	6.7
CO-like	22	3	13.6	6	27.3
CPP	12	1	8.3	1	8.3
DBB	20	6	30.0	2	10.0
Dof	71	17	23.9	7	9.9
E2F/DP	14	2	14.3	2	14.3
EIL	10	0	0.0	0	0.0
ERF	245	31	12.7	50	20.4
FAR1	49	5	10.2	1	2.0
G2-like	94	24	25.5	18	19.1
GATA	50	10	20.0	5	10.0
GeBP	8	4	50.0	1	12.5
GRAS	104	24	23.1	17	16.3
GRF	21	0	0.0	1	4.8
HB-other	18	4	22.2	2	11.1
HB-PHD	6	2	33.3	0	0.0
HD-ZIP	85	15	17.6	15	17.6
HRT-like	1	1	100.0	0	0.0
HSF	48	5	10.4	22	45.8
LBD	62	9	14.5	14	22.6
LFY	1	0	0.0	0	0.0
LSD	8	2	25.0	1	12.5
MIKC_MADS	53	11	20.8	7	13.2
M-type_MADS	14	1	7.1	2	14.3
MYB	241	32	13.3	44	18.3
MYB_related	139	19	13.7	26	18.7
NAC	167	23	13.8	33	19.8
NF-X1	5	0	0.0	0	0.0
NF-YA	21	6	28.6	5	23.8
NF-YB	29	4	13.8	5	17.2
NF-YC	17	4	23.5	1	5.9
Nin-like	21	3	14.3	7	33.3
RAV	4	3	75.0	1	25.0
S1Fa-like	4	1	25.0	0	0.0
SAP	1	0	0.0	0	0.0
SBP	38	5	13.2	4	10.5
SRS	21	7	33.3	3	14.3
STAT	1	1	100.0	0	0.0
TALE	63	12	19.0	3	4.8
TCP	44	4	9.1	9	20.5
Trihelix	67	8	11.9	10	14.9
VOZ	6	1	16.7	0	0.0
Whirly	7	1	14.3	0	0.0
WOX	18	2	11.1	0	0.0
WRKY	171	31	18.1	38	22.2
YABBY	6	0	0.0	0	0.0
ZF-HD	24	3	12.5	3	12.5
Total	3017	513	17.0	491	16.3

### Trimethylation of H3K27 Under Salt Stress in Soybean

**Figure 4:**
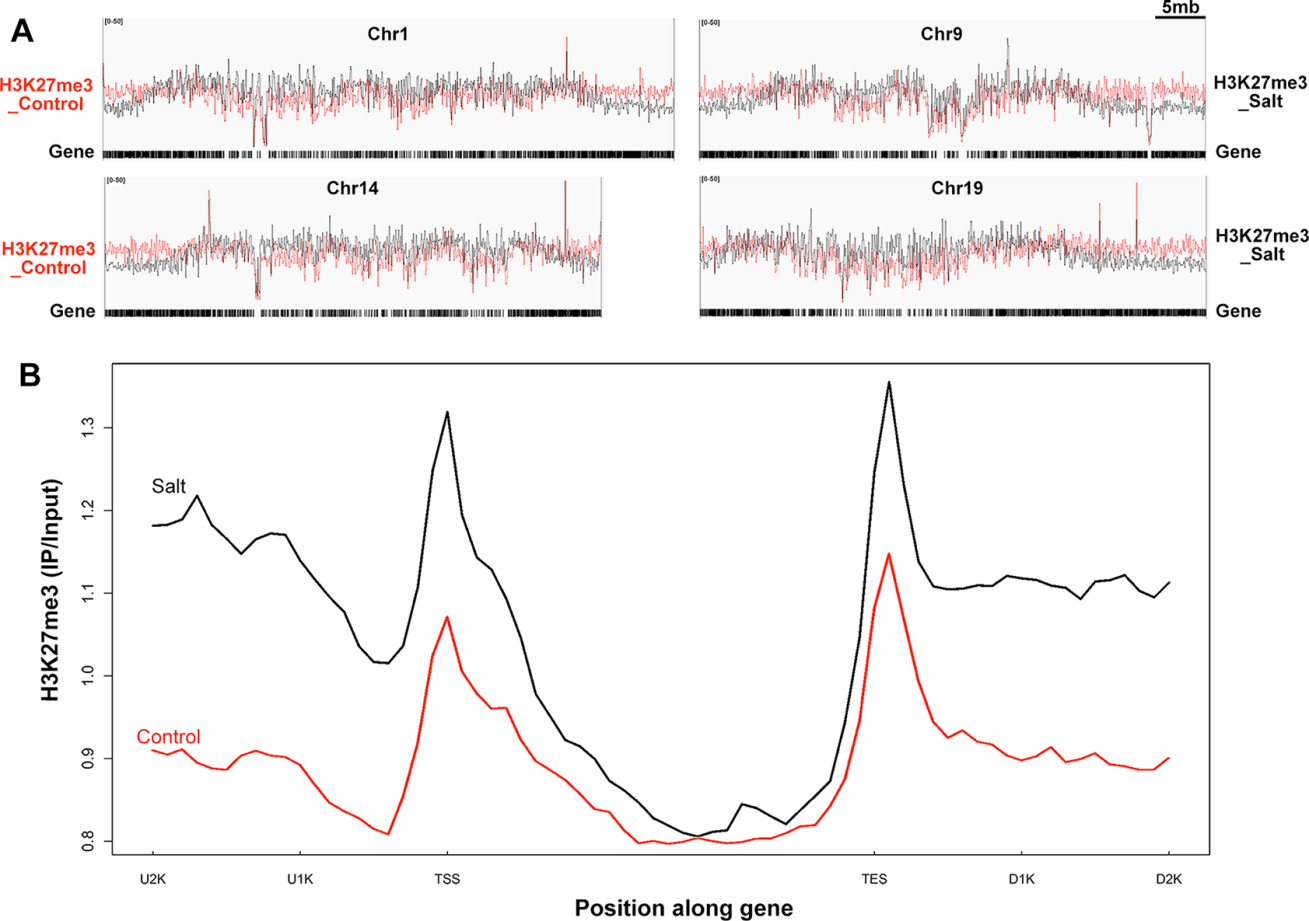
Genome-wide H3K27me3 modification pattern in control and salt-treated soybean. **(A)** Chromosomal distribution of H3K27me3 modification sites on the randomly selected 4 soybean chromosomes. Y-axis represents the input signals for the immunoprecipitation of H3K27me3 in control on the left side (H3K27me3_Control) and salt-treated soybean on the right side (H3K27me3_Salt). The comparison of H3K27me3 marked in control (red) and salt (black) plants were shown on all chromosomes. Chr and 5mb represent chromosome and 5 megabase, respectively. Gene models shown at the bottom. **(B)**The H3K27me3 patterns of all trimethylated genes in control and salt-treated soybean. The gene sequences were aligned at the transcription start site (TSS) and average signals of the H3K27me3 enrichment 2kb upstream (U2K), 3kb gene body, and 2kb downstream of the TES (D2K) were plotted.

Trimethylated histone H3 at lysine residues 27 (H3K27me3) has been detected in many organisms, including *Arabidopsis*, rice, and maize (Butenko and Ohad, 2011). It is a hallmark of gene silencing (Schubert et al., 2006; Zheng and Chen, 2011). However, whether this repressive mark is involved, and to what extent, in salt stress response in soybean is unknown. To determine the alteration of chromatin dynamics and transcriptional apparatus that respond to environmental changes, we applied ChIP-seq to monitor the changes of H3K27me3 levels at genome-wide scale under salt stress treatment in soybean (**Figure 4**). ChIP-seq was performed by using an antibody specifically recognizing H3K27me3 (Pu et al., 2013; Xu et al., 2018), and the precipitated DNAs were then sequenced. After sequencing, we obtained about 50 million of clean reads with 75–85% of the reads that could be mapped to the soybean genome (**Supplementary Table 4**). Verification of ChIP-seq results using Pearson correlation analysis showed statistically significant correlation coefficients among the biological replicates for each sample (**Supplementary Figure 2**). Genomic regions associated with H3K27me3 modification were identified by using MACS software (Zhang et al., 2008). The peak distributions of ChIP-seq are similar and average length of peaks is around 700 bp in samples of control and salt-treated plants (**Supplementary Figures 3A, B**).

The MACS peak finding program identified thousands of H3K27me3 enriched peaks in control and salt-treated samples (p < 10^−3^) across the whole chromosome (**Figure 4A** and **Supplementary Figure 4**), which correspond to 1,707 and 746 annotated genes, respectively (**Supplementary Figure 3B**). As reported previously in *Arabidopsis* (Kim et al., 2012b; Xu et al., 2018), H3K27me3 peaks tend to be broad, often covering the entire transcriptional unit, hence we used a very strict statistical cutoff for peak identification. In control plants, the 1,707 genes were termed K27 genes in the next analysis vs. *de novo*_K27 genes (**Supplementary Tables 5** **and** **6**). We plotted the average H3K27me3 signal of the 1,707 K27 genes across the 7 kb region surrounding the transcription start site (TSS) and the transcription end site (TES) in the control soybean (**Figure 4B**). Similar to that of *Arabidopsis*, a broad H3K27me3 enrichment covers the entire transcriptional unit with the strongest signal around the TSS region, whereas the H3K27me3 signal gradually declined towards the 3’ end and increased around the TES region, suggesting the conservation and divergence of epigenetic patterns across plant species (**Figure 4B**). We then checked the histone modification pattern of 746 H3K27me3 marked genes in salt-treated soybean which remained remarkably similar to that in control plants (**Figure 4B** and **Supplementary Table 6**). Compared to H3K27me3 pattern in control plants, the epigenetic marks of H3K27me3 showed a greater enrichment in salt-stressed samples (**Figure 4B**), suggests that stress caused changes in chromatin structure and histone modification which accompany changes in gene expression in response to abiotic stresses.

### Relationship Between Changes in H3K27me3 and Gene Expression Under Salt Stress

H3K27me3 has been proposed to be correlated with gene silencing in many organisms (Pu and Sung, 2015). So we questioned if H3K27me3 modification is correlated with a different expression level under salt stress. We combined the specific H3K27me3 modification datasets with our DEGs to identify the relationship between H3K27me3 modification and different expression levels. We found that 829 of the 1,707 H3K27me3 specifically modified genes were not expressed in both of control and salt samples, despite some of them were not trimethylated (**Table 2**) which may be caused by our criteria used for analyzing the RNA-seq data (see Materials and methods section) as reported in our previous study (Xu et al., 2018). It is also possibly caused by the fact that not all genes expressed in soybean roots. By excluding those non-expressed genes, only 878 (51%) expressed genes were trimethylated on H3K27 in the control and salt datasets (**Table 2**). These specific H3K27me3 genes were then checked for the expression level changes in the corresponding treatment, and the numbers of up- and down-regulated genes in each of the specific H3K27me3 modification datasets were further analysed (**Table 2**). Under salt stress treatment, 170 of 336 K27 genes (50.6%) were up-regulated (**Table 2**). Statistical tests of the genome-wide relationship between reduced H3K27me3 and transcriptional deregulation (**Table 2**) in the salt-treated plants showed that 50.6% (*p*-value = 1.03x10^−15^) of up-regulated genes also had reduced H3K27me3 levels.

**Table 2:** The gene expression changes of methylated H3K27me3 genes in salt-treated soybean.

Total number of genes investigated	Number of genes with expression in RNA-seq data	Number of genes with decreased K27	Up-regulated expression		
			Number	%	*p*-value
1,707	878	336	170	50.6	1.03 x 10^−15^

**Figure 5:**
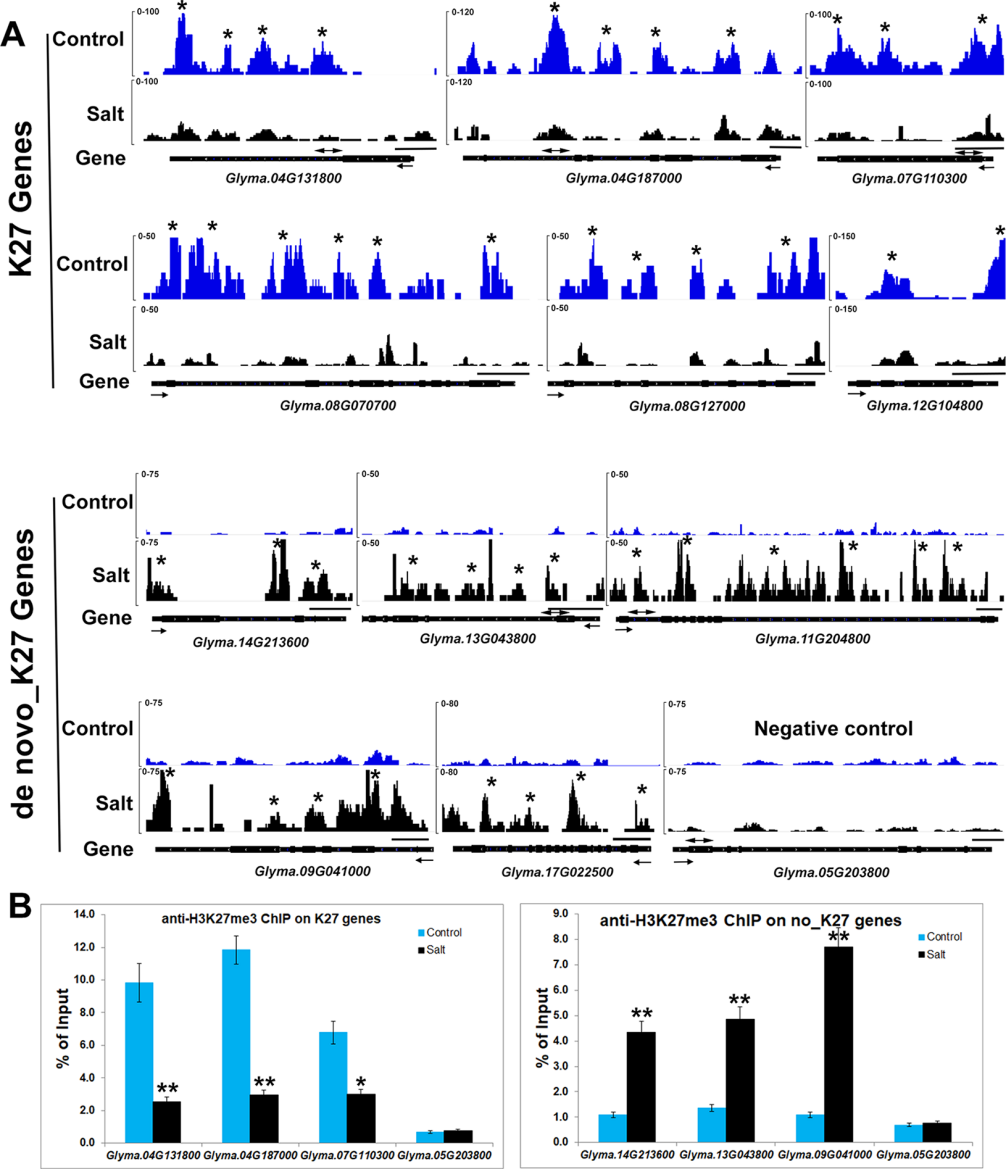
Salt stress affects histone methylation at salt stress gene loci in soybean. **(A)** H3K27me3 patterns of K27 and *de novo*_K27 genes from ChIP-seq data in control and salt-treated soybeans. Gene models are shown at the bottom including 5’ UTR (medium black line), exon (black box), intron (thin black line) and 3’ UTR (medium black line). The arrow indicates transcriptional direction. The black line above gene model indicates 500bp. The “*” indicates that the MACS_peak with the statistical identification each sample using MACS with the default 10^−5^ *p*-value cutoff. **(B)** ChIP-qPCR analysis of H3K27me3 levels at the salt stress genes in soybean under salt stress by using *Glyma.05G203800* (*Tubulin*) gene as the negative control. ChIP-qPCR results are expressed as a percentage of input DNA, with error bars representing SD. Primers (double arrowheads) correspond to the gene regions shown in **(A)**. Significant differences from the control (Student’s t test) are marked with asterisks (**P <0.01).

We noticed that H3K27me3 is associated with expression changes of specific salt stress genes which likely contribute to response to environmental changes. Eleven genes, *Glyma.17G006800*, *Glyma.08G070700*, *Glyma.08G127000*, *Glyma.15G252200*, *Glyma. 12G104800*, *Glyma.07G110300*, *Glyma.17G223600*, *Glyma.20G021200*, *Glyma.04G13180*0, *Glyma.04G187000*, *Glyma.20G072600* were significantly up-regulated in salt treated samples (**Supplementary Table 3**). Six salt stress genes, *Glyma.08G070700*, *Glyma.08G127000*, *Glyma.12G104800*, *Glyma.07G110300*, *Glyma. 04G13180*0, *Glyma.04G187000*, showed lower H3K27me3 levels and higher mRNA expression levels after salt treatment (**Figure 3** and **Figure 5A**). To confirm the ChIP-seq results, we performed ChIP-qPCR on three selected salt response genes, *Glyma.07G110300*, *Glyma.04G13180*0, *Glyma.04G187000*, that showed enhanced expression levels under stress, using the un-methylated *Glyma.05G203800* (*Tubulin*) gene as the negative control (**Figure 5B**). We found that these salt response genes had much higher H3K27me3 levels in the control plants which decreased greatly under salt stress indicating that salt stress can remove the deposition of repressive chromatin marks at these loci during stress treatment (**Figure 5**). These results show that plants respond to the environmental changes through the transcriptional machinery in which transcription was turned on or shut down by changing the mode of histone modifications between activation and inactivation on all of stress response genes.

### Salt Stress Causes *de novo* Histone Methylation and Gene Silencing Under Salt Stress in Soybean

Surprisingly, we found that there were only 5 of 878 K27genes (0.5%) with increased H3K27me3 marks in salt treatment. We then asked what happened to the *de novo*_K27 genes after salt stress treatment. It has been reported that *de novo* methylation can occur in a locus-specific manner during development in yeast, plant and animals (Ooi et al., 2007; Bouyer et al., 2011; Morselli et al., 2015; Stewart et al., 2015). Among 746 H3K27me3 marked genes in salt-treated soybean, we found 651 genes appeared to be marked *de novo* H3K27me3 in salt-treated plants which mainly contributed to the greater H3K27me3 pattern in salt-treated plant (**Supplementary Table 6**).

Among 651 genes, there are 294 genes with expression data in our RNA-seq analysis (**Table 3**). Statistical tests of the genome-wide relationship between increased H3K27me3 and transcriptional deregulation (**Table 3**) in the salt-treated plants showed that 33.7% (*p*-value = 1.9x10^−31^) of down-regulated genes also had increased H3K27me3 levels. Some of these genes are stress-responsive genes such as *Glyma.14G213600*, *Glyma.11G204800*, *Glyma.09G041000*, *Glyma.13G043800* and *Glyma.17g022500* (**Figures 3** and **5**). Although the gain of H3K27me3 is not associated with all down-regulated genes, it contributed the greater level of H3K27me3 modification in salt stress condition compared to that in control plants.

**Table 3:** The gene expression changes of *de novo* methylated H3K27me3 genes in salt-treated soybean.

Total number of genes investigated	Number of genes with expression in RNA-seq data	Down-regulated expression		
		Number	%	*p*-value
651	294	99	33.7	1.9x10^-31^

### Changes in Histone Methylation and Demethylation Contribute to Changes in H3K27me3 Modification Levels Under Salt Stress in Soybean

**Figure 6:**
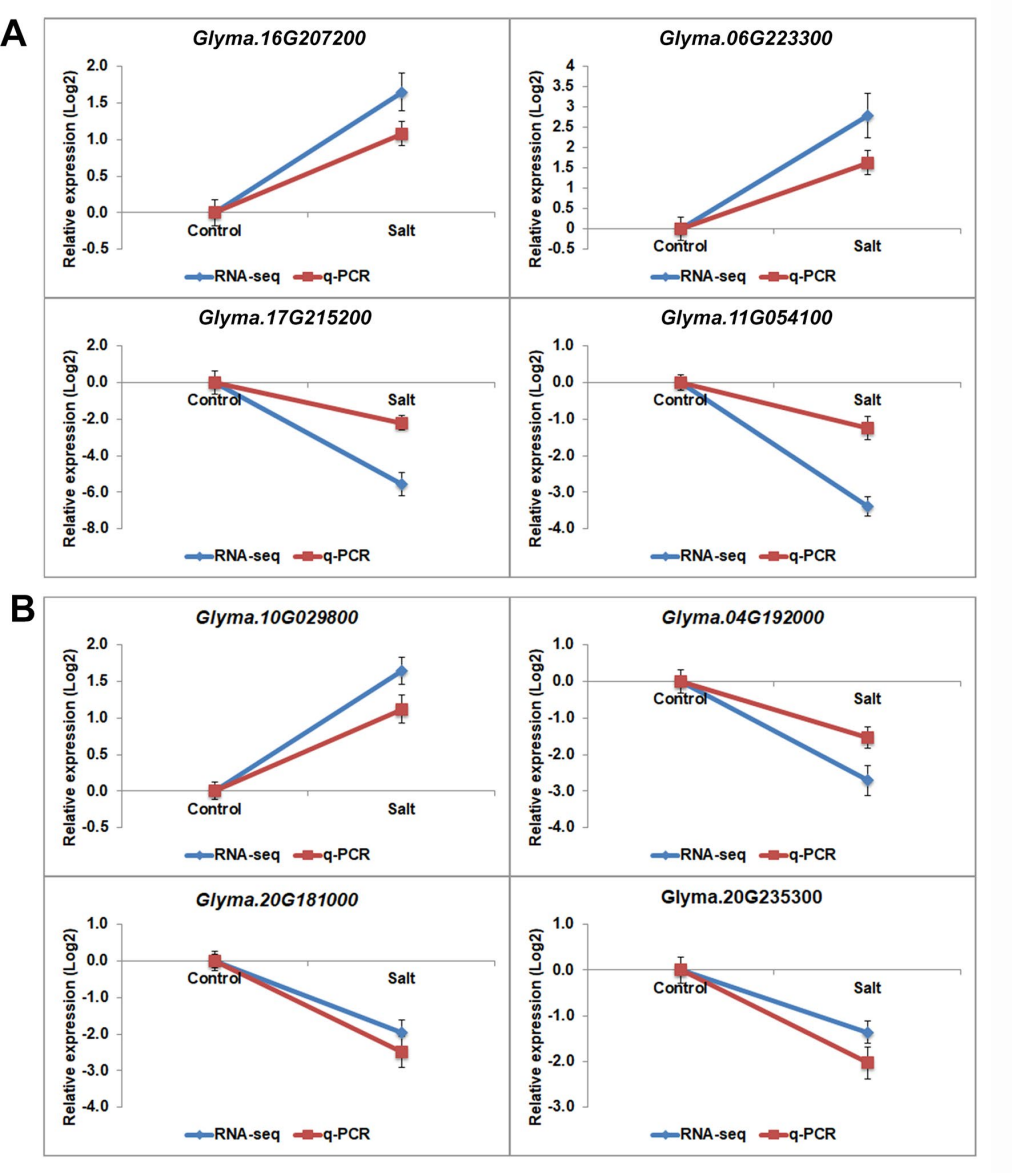
The gene expression pattern of selected histone modifier genes analyzed by RNA-seq and q-PCR. mRNA expression levels of 4 histone methyltransferases **(A)** and histone demethylasesgenes **(B)** with differential expression levels in salt-treated soybean compared to control plants. Graphs show the relative expression levels analyzed by RNA-seq and by qPCR which normalized to a *Tubulin* (*Glyma.05G203800*) reference gene. Error bars represent standard deviation (SD).

The H3K27 was trimethylated by histone methyl transferases (HMTs) and demethylated by HDMs (Papp and Muller, 2006; Horton et al., 2010; Pu and Sung, 2015). To explore how histone modifications were regulated when under salt stress in soybean, we used RNA-seq data to investigate the candidate causal genes of methyltransferase or demethylase for salt response. We identified 43 HMT proteins from Soybase according to the protein sequence homology with *Arabidopsis* HMTs (Grant et al., 2010). Specifically, 9 soybean genes which are homologous to *Arabidopsis* known methyltransferase genes *CURLY LEAF (CLF)*, *ATX* and *SDG*, were down-regulated, and 2 genes are up-regulated with significant *p*-value in salt-treated plants (**Table 4**). CLF has been well characterized in *Arabidopsis* to work specifically as H3K27 methyltransferases (Katz et al., 2004; Schatlowski et al., 2010). ATX and SDG proteins were known to methylate H3K4 and limit deposition of H3K27me3 on target loci (Ding et al., 2007; Carles and Fletcher, 2009; Grini et al., 2009; Berr et al., 2010; Guo et al., 2010; Tang et al., 2012; Yao et al., 2013). To verify the RNA-seq results, we examined the RNA levels of 4 selected methyltransferase genes, *Glyma.16G207200*, *Glyma.06G223300*, *Glyma.17G215200* and *Glyma.11G054100*, by qPCR (**Figure 6A**). The expression levels determined by qPCR and RNA-seq were highly correlated (**Figure 6A**), indicating that the results obtained by the independent methods are consistent. Therefore, these soybean proteins may function as methyltransferase to alter histone modifications of target loci for salt response.

**Table 4:** Expression profile of histone methyltransferases in soybean.

Gene	*Arabidopsis* homologues and Annotation		Log_2_ FC	*p-value*
*Glyma.17G215200*	*AT1G05830*	ATX2|trithorax-like protein 2	−5.58	1.89E-05
*Glyma.16G100200*	*AT4G13460*	SDG22, SUVH9, SET22|SU(VAR)3-9 homolog 9	−3.83	0.068396
*Glyma.11G054100*	*AT2G23380*	CLF, ICU1, SDG1, SET1|SET domain-containing protein	−3.38	2.52E-06
*Glyma.13G186800*	*AT5G04940*	SUVH1|SU(VAR)3-9 homolog 1	−2.60	0.001074
*Glyma.07G056000*	*AT3G61740*	SDG14, ATX3|SET domain protein 14	−2.28	0.003279
*Glyma.06G151500*	*AT5G09790*	ATXR5, SDG15|ARABIDOPSIS TRITHORAX-RELATED PROTEIN 5	−2.14	0.032018
*Glyma.01G188000*	*AT2G23380*	CLF, ICU1, SDG1, SET1|SET domain-containing protein	−1.84	0.012066
*Glyma.15G224400*	*AT1G73100*	SUVH3, SDG19|SU(VAR)3-9 homolog 3	−1.81	0.020458
*Glyma.20G005400*	*AT4G13460*	SDG22, SUVH9, SET22|SU(VAR)3-9 homolog 9	−1.71	0.028072
*Glyma.12G196800*	*AT4G15180*	SDG2, ATXR3|SET domain protein 2	−1.67	0.007215
*Glyma.13G306800*	*AT3G21820*	ATXR2, SDG36|histone-lysine N-methyltransferase ATXR2	−1.41	0.643551
*Glyma.02G095600*	*AT5G24330*	ATXR6, SDG34|ARABIDOPSIS TRITHORAX-RELATED PROTEIN 6	−1.27	0.79442
*Glyma.04G236500*	*AT5G53430*	SDG29, SET29, ATX5|SET domain group 29	−1.21	0.127662
*Glyma.11G038000*	*AT2G22740*	SUVH6, SDG23|SU(VAR)3-9 homolog 6	−1.20	0.147649
*Glyma.19G124100*	*AT1G73100*	SUVH3, SDG19|SU(VAR)3-9 homolog 3	−1.11	0.179543
*Glyma.07G157400*	*AT4G13460*	SDG22, SUVH9, SET22|SU(VAR)3-9 homolog 9	−0.96	0.173577
*Glyma.15G158500*	*AT5G42400*	ATXR7, SDG25|SET domain protein 25	−0.78	0.263644
*Glyma.04G125500*	*AT5G04940*	SUVH1|SU(VAR)3-9 homolog 1	−0.66	0.353851
*Glyma.16G024900*	*AT3G61740*	SDG14, ATX3|SET domain protein 14	−0.55	0.580097
*Glyma.03G119900*	*AT1G73100*	SUVH3, SDG19|SU(VAR)3-9 homolog 3	−0.46	0.436713
*Glyma.13G305000*	*AT4G15180*	SDG2, ATXR3|SET domain protein 2	−0.46	0.467736
*Glyma.09G156500*	*AT2G44150*	ASHH3, SDG7|histone-lysine N-methyltransferase ASHH3	−0.28	0.645242
*Glyma.02G012100*	*AT4G02020*	EZA1, SWN, SDG10|SET domain-containing protein	−0.23	0.840641
*Glyma.10G222800*	*AT1G76710*	ASHH1|SET domain group 26	−0.18	0.806477
*Glyma.19G066800*	*AT4G27910*	ATX4, SDG16|SET domain protein 16	−0.11	0.871815
*Glyma.10G012600*	*AT4G02020*	EZA1, SWN, SDG10|SET domain-containing protein	−0.04	0.953137
*Glyma.03G215600*	*AT3G61740*	SDG14, ATX3|SET domain protein 14	0.06	0.943206
*Glyma.06G301900*	*AT4G15180*	SDG2, ATXR3|SET domain protein 2	0.18	0.765767
*Glyma.04G245400*	*AT1G77300*	EFS, SDG8, CCR1, ASHH2, LAZ2|histone methyltransferases(H3-K4 specific);histone methyltransferases(H3-K36 specific)	0.26	0.651076
*Glyma.04G214600*	*AT5G09790*	ATXR5, SDG15|ARABIDOPSIS TRITHORAX-RELATED PROTEIN 5	0.29	0.887053
*Glyma.11G040100*	*AT2G22740*	SUVH6, SDG23|SU(VAR)3-9 homolog 6	0.37	0.869301
*Glyma.12G195700*	*AT3G21820*	ATXR2, SDG36|histone-lysine N-methyltransferase ATXR2	0.38	0.54887
*Glyma.01G204900*	*AT2G22740*	SUVH6, SDG23|SU(VAR)3-9 homolog 6	0.40	0.514298
*Glyma.18G282700*	*AT1G05830*	ATX2|trithorax-like protein 2	0.40	0.524246
*Glyma.12G102400*	*AT4G15180*	SDG2, ATXR3|SET domain protein 2	0.45	0.446104
*Glyma.09G052200*	*AT5G42400*	ATXR7, SDG25|SET domain protein 25	0.49	0.445646
*Glyma.20G168900*	*AT1G76710*	ASHH1|SET domain group 26	0.55	0.376492
*Glyma.06G117700*	*AT1G77300*	EFS, SDG8, CCR1, ASHH2, LAZ2|histone methyltransferases(H3-K4 specific);histone methyltransferases(H3-K36 specific)	0.60	0.345329
*Glyma.08G258500*	*AT1G05830*	ATX2|trithorax-like protein 2	0.76	0.545905
*Glyma.18G285900*	*AT5G24330*	ATXR6, SDG34|ARABIDOPSIS TRITHORAX-RELATED PROTEIN 6	1.08	0.247913
*Glyma.16G207200*	*AT2G44150*	ASHH3, SDG7|histone-lysine N-methyltransferase ASHH3	1.65	0.024473
*Glyma.01G202700*	*AT2G22740*	SUVH6, SDG23|SU(VAR)3-9 homolog 6	2.64	0.077437
*Glyma.06G223300*	*AT4G30860*	ASHR3, SDG4|SET domain group 4	2.79	0.003304

Jumonji C (JmjC) proteins are known to demethylate all of the mono-, di and trimethylated lysines of histones (Chen et al., 2011). There are over 20 JmjC proteins have been discovered in *Arabidopsis* which are able to demethylate lysine H3K4, H3K9, H3K27, and H3K36. We checked the expression pattern of 21 JmjC proteins in salt-treated soybeans (**Table 5**). Interestingly, we found that 3 of JmjC proteins were down-regulated, and 1 was up-regulated (**Table 5** and **Figure 6B**). 2 of down-regulated genes, *Glyma.04G192000* and *Glyma.20G181000*, are homologues to *Arabidopsis* *Early flowering 6* (*REF6*) and *Relative of ELF6* (*ELF6*) which are known demethylases to mediate histone methylation (Yu et al., 2008; Lu et al., 2011). These results indicated the involvement of histone modifiers in the changing H3K27me3, subsequently transcript levels during salt stress.

**Table 5:** Expression profile of the histone demethylases in soybean.

Gene	*Arabidopsis* homologues and Annotation		Log_2_ FC	*p*-value
*Glyma.04G192000*	*AT3G48430*	REF6|relative of early flowering	−2.71	0.0017
*Glyma.20G181000*	*AT5G04240*	ELF6|Zinc finger (C2H2 type) family protein/transcription factor jumonji (jmj) family prote	−1.97	0.0032
*Glyma.04G185900*	*AT5G63080*	2-oxoglutarate (2OG) and Fe(II)-dependent oxygenase superfamily protein	−1.87	0.1304
*Glyma.06G174000*	*AT3G48430*	REF6|relative of early flowering 6	−1.48	0.1951
*Glyma.19G064000*	*AT1G62310*	Transcription factor jumonji (jmjC) domain-containing protein	−1.43	0.0825
*Glyma.20G235300*	*AT1G09060*	Zinc finger, RING-type;Transcription factor jumonji/aspartyl beta-hydroxylas	−1.36	0.0271
*Glyma.14G159400*	*AT1G11950*	Transcription factor jumonji (jmjC) domain-containing protein	−0.99	0.5115
*Glyma.12G055000*	*AT3G20810*	JMJD5|2-oxoglutarate (2OG) and Fe(II)-dependent oxygenase superfamily protei	−0.25	0.6778
*Glyma.11G130600*	*AT3G20810*	JMJD5|2-oxoglutarate (2OG) and Fe(II)-dependent oxygenase superfamily protein	−0.03	0.9667
*Glyma.10G209600*	*AT5G04240*	ELF6|Zinc finger (C2H2 type) family protein/transcription factor jumonji (jmj) family protein	0.13	0.8604
*Glyma.19G068800*	*AT4G00990*	Transcription factor jumonji (jmjC) domain-containing protein	0.16	0.7779
*Glyma.02G144300*	*AT5G19840*	2-oxoglutarate (2OG) and Fe(II)-dependent oxygenase superfamily protein	0.16	0.8740
*Glyma.09G207400*	*AT5G46910*	Transcription factor jumonji (jmj) family protein/zinc finger (C5HC2 type) family protein	0.26	0.6988
*Glyma.11G023700*	*AT1G63490*	transcription factor jumonji (jmjC) domain-containing protein	0.36	0.5608
*Glyma.20G104900*	*AT4G00990*	Transcription factor jumonji (jmjC) domain-containing protein	0.48	0.6567
*Glyma.07G263200*	*AT1G11950*	Transcription factor jumonji (jmjC) domain-containing protein	0.60	0.4016
*Glyma.10G284500*	*AT4G00990*	Transcription factor jumonji (jmjC) domain-containing protein	0.74	0.4306
*Glyma.10G153000*	*AT1G09060*	Zinc finger, RING-type;Transcription factor jumonji	0.88	0.1398
*Glyma.01G219800*	*AT1G63490*	transcription factor jumonji (jmjC) domain-containing protein	0.92	0.1816
*Glyma.01G014700*	*AT5G46910*	Transcription factor jumonji (jmj) family protein/zinc finger (C5HC2 type) family protei	1.36	0.0890
*Glyma.10G029800*	*AT5G19840*	2-oxoglutarate (2OG) and Fe(II)-dependent oxygenase superfamily protein	1.65	0.0037

### Overexpression of One Soybean Gene Enhances the Salt Tolerance in Transgenic *Arabidopsis*

**Figure 7:**
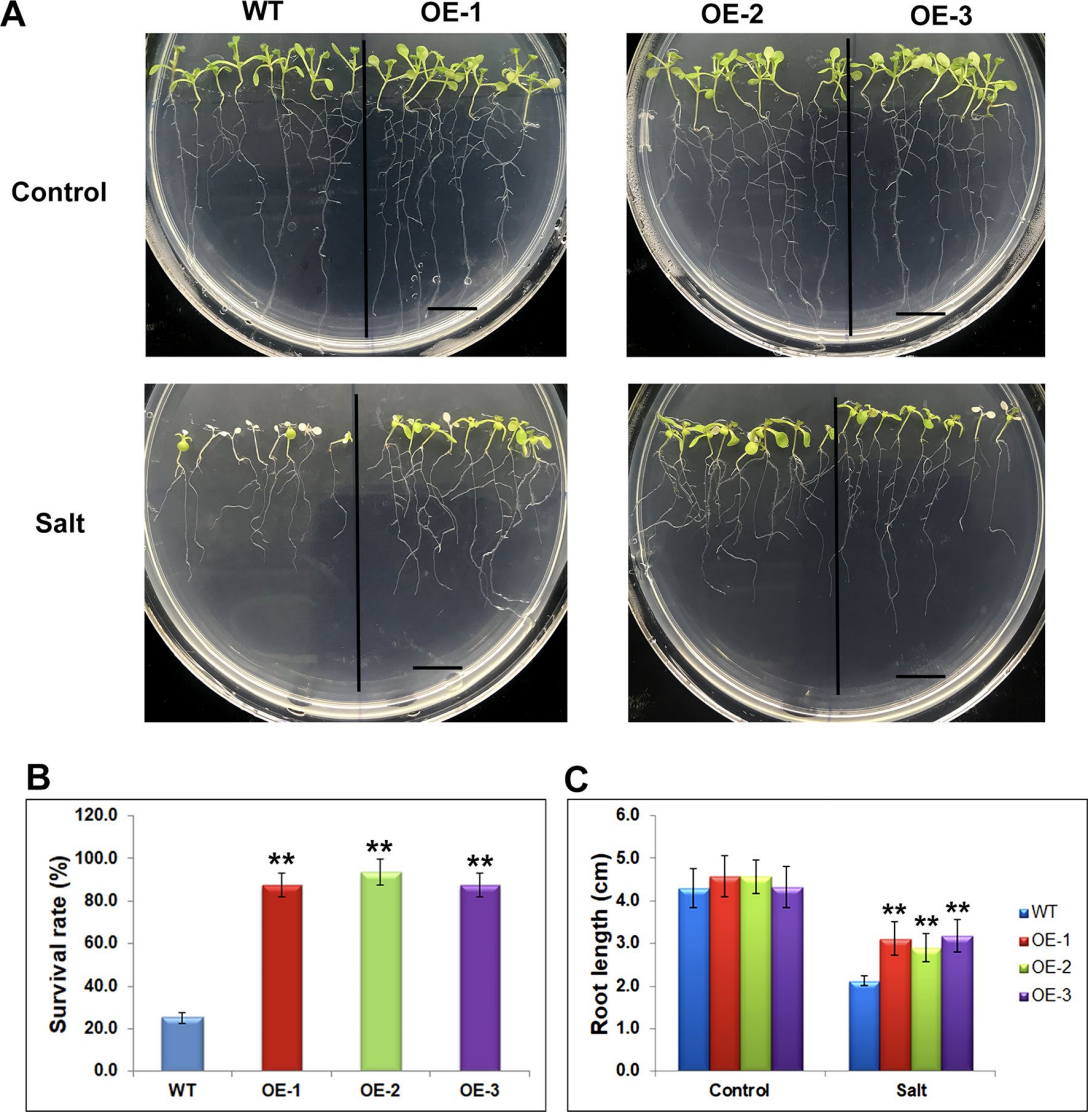
Phenotypes of *Glyma.17g022500* transgenic plants under salt stress. **(A)** Salt tolerance assay of the *Glyma.17g022500* overexpression (OE) lines, OE-1, OE-2, OE-3 plants. WT, OE-1, OE-2 and OE-3 seedlings at 5 DAG were transferred from MS medium to MS medium containing 150 mM salt and grown for an additional 5 days. Bar = 1 cm. **(B)** Survival rate of the plants in **(A)** under salt stress. The data presented are the mean ± SD (n = 50). **(C)** Root length of seedlings grown on medium with and without salt. Root length was measured after 5 days of growth on MS or MS with salt (n = 50). Significant differences from the WT (Student’s t test) are marked with asterisks (**P <0.01).

The differentially expressed genes identified through RNA-seq were considered to be preferentially genes involved in abiotic stress responses, suggesting their stress regulation in the soybean plants. To investigate whether these genes would affect the stress response, we selected one of mis-regulated genes, *Glyma.17G022500* and studied its effect on salt tolerance or sensitivity in *Arabidopsis*. The transgenic *Arabidopsis* plants containing *Glyma.17G022500* under the control of the *CaMV35S* promoter in the *pCAMBIA1301* vector were generated. Independent transgenic lines were obtained by Hygromycin-resistance selection and confirmed by genotyping PCR. The homozygous T3 generation of three independent overexpression lines, namely OE-1, OE-2, OE-3, and the control line (WT) were used for further analysis (**Supplementary Figure 5**). To avoid adverse effects of salt treatment on germination, we transferred 5 days’ seedlings of WT, OE-1, OE-2, and OE-3, from MS plates to MS medium containing 150 mM of salt and grew them for an additional 5 days under SD conditions. After 5 days salt stress, the transgenic *Glyma.17G022500* lines displayed a higher salt tolerance than the WT plants (**Figure 7**). As shown in **Figure 7A**, the WT plants became severely wilted and impaired with white cotyledons and leaves after salt stress. However, the transgenic *Glyma.17G022500* lines showed more open, green leaves in all three independent lines (**Figure 7A**). The *Glyma.17G022500* transgenic lines displayed significantly higher survival rate than the WT plants after salt treatment (**Figure 7B**). Furthermore, we found that the roots in the transgenic lines grew longer than that in the WT plants on salt plates (**Figure 7C**). These results indicate that overexpression of *Glyma.17G022500* enhances *Arabidopsis* salt stress tolerance which confirmed our RNA-seq results.

## Discussion

The soybean gene methylation pattern is characteristic of plant methylation pattern. Here, we investigated the modification profiles of H3K27me3 after salt stress treatment in soybean. H3K27me3 was detected mainly in TSS and TES regions and 1,707 annotated genes were identified with H3K27me3 marks (**Figure 4**), which displayed the conservation and divergence of epigenetic patterns to previous studies in *Arabidopsis* (Zhang et al., 2007; Kim et al., 2012b). We further analyzed K27, *de novo*_K27 genes as well as DEGs and revealed different dynamic changes in H3K27me3 profiles taking place upon salt stress. The specific H3K27me3 patterns including *de novo* methylation at up-regulated and down-regulated genes were identified during the stress treatment. In addition, a comprehensive overview of the histone modifiers were identified which may regulate differential H3K27me3 modification leading to activation or inactivation of gene expression during salt stress in soybean.The certain proportion of H3K27me3-modified genes without expression support also implies that the H3K27me3 level may be associated with expression levels of a subset of genes in soybean genome by working together with other factors, such as the HMT, SDG proteins which bind the H3K27me3 site (Papp and Muller, 2006; Schuettengruber et al., 2011; Thorstensen et al., 2011).

The differentially expressed genes identified in this study were considered to be the key genes involved in the stress response mechanism in the plants. Some of them have been shown to be related to salt response in soybean. For example, *GmSALT3/GmCHX1* (*Glyma.03G171600*) which is a gene associated with salt tolerance with great potential for soybean improvement showed down-regulated expression pattern after salt treatment (Guan et al., 2014; Qi et al., 2014; Liu et al., 2016). The Na +/H+ antiporter gene *GmNHX1* (*Glyma.20G229900*) which can enhance salt tolerance of soybean roots (Li et al., 2006; Sun et al., 2006; Yang et al., 2017). A soybean glycogen synthase kinase 3 gene which can enhance tolerance to salt was up-regulated in salt treated soybean in this study (Wang et al., 2018). Other known salt responsive genes identified through RNA-seq analysis (Zeng et al., 2019) such as *Glyma.02G228100*, *Glyma.04G180400*, *Glyma.03G226000*, *Glyma.08G189600*, *Glyma.02G228100* et al., were also identified in this study. We also identified new candidate genes for salt response in soybean. For example, the gene on Chr. 7, *Glyma.07G110300*, which was up-regulated in the salt-treated plants (**Figure 3**) was annotated as “UDP-glucosyltransferase superfamily protein” in this study, which was in agreement with earlier observations that the glucosyltransferase modulates abiotic stress tolerance in *Arabidopsis* (Tognetti et al., 2010; Liu et al., 2015). The gene *Glyma.04G131800*, which was annotated as “prohibitin-3, mitochondrial”, was also up-regulated in the salt-treated soybeans (**Figure 3**). The members of prohibitin family acted in stress response (Wang et al., 2010; Seguel et al., 2018). The gene *Glyma.04G187000* encodes a histone deacetylase which is a histone modifier with direct function in regulation of stress response in plants (Chen and Wu, 2010; Chen et al., 2010; Luo et al., 2012b; Zheng et al., 2016). Overexpression of one mis-regulated gene, *Glyma.17G022500*, in *Arabidopsis* resulted in higher survival rates than those in WT lines under salt stress (**Figure 7B**), and the resistance to salt was significantly different (**Figure 7**). Therefore, we conclude that *Glyma.17G022500* has an important effect on resistance to salt stress. This analysis of gene expression patterns between control and salt plants provides a number of candidate genes which might be directly or indirectly involved in the stress response trait. The further genetic analysis and transformation experiments could be used to confirm their roles in salt stress response in the soybean genotypes.

The repression of genes in development mediated by H3K27me3 modification is a highly conserved mechanism in both plants and animals. There are several thousand genes, ∼ 20% of all transcribed genes, are marked by such modifications in *Arabidopsis* (Zhang et al., 2007; Hennig and Derkacheva, 2009; Lu et al., 2011; Kim et al., 2012b; Xu et al., 2018). Here, there are only ∼2,000 (5%) genes identified as H3K27me3 marked genes in soybean which is lower than the average percentage in *Arabidopsis*. Our results showed that H3K27me3 was correlated with only small parts of genome-wide transcript changes of mis-regulated genes during salt stress response in soybean (**Tables 2** and **3**). This may be due to H3K27me3 not being the only repressive histone modification marks for gene silencing in soybean since other repressive or active histone modification marks may play a vital role in regulating gene expression in response to stress (Chinnusamy and Zhu, 2009; Kim et al., 2012a; Liew et al., 2013; Zong et al., 2013; Yang et al., 2014; Liu et al., 2018). It has been reported that many histone modification marks such as active marks: H3K27ac, H3K4me3, H3K36me3, H3K9ac, and repressive marks: H3K9me3, H2K119ub (Bratzel et al., 2010; Gu et al., 2013; Yang et al., 2014; Xu et al., 2016), are known to be positively or negatively correlated with active or silencing transcription in plants. Indeed, here we found the gene of *Glyma.04G187000* which encodes a histone deacetylase was up-regulated in soybean under salt stress (**Figure 3**), suggesting that it may regulate gene expression through histone acetylation.

**Figure 8:**
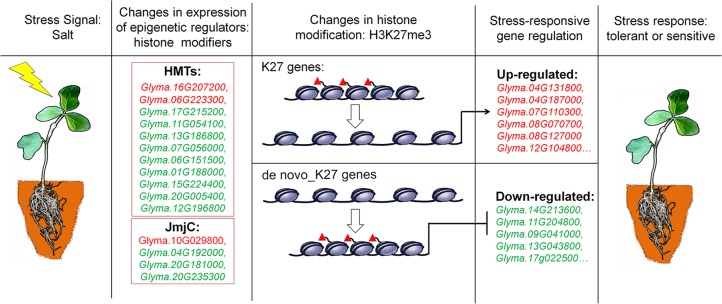
A proposed model for epigenetic regulation of stress response in soybean. Salt stress signals induce changes in the expression of epigenetic regulators of histone modifiers such as histone methyltransferases (HMTs) and histone demethylases (JmjC). These epigenetic regulators specifically modify histone modifications of H3K27me3 on K27 or *de novo*_k27 genes which lead to the expression changes of salt-responsive genes. The different behavior of methylation marks during the response process illustrates that they have distinct roles in the transcriptional response of implicated genes. Genes up-regulated in salt-treated soybean are marked red, and down-regulated are green respectively. Red triangles represent H3K27me3 marks.

Chromatin accessibility is defined as the availability of DNA sequences for molecular interactions, typically mediated through by DNA binding factors and nucleosomes that are the major factors of chromatin accessibility (van Steensel, 2011). Nucleosome-free regions have been observed in many organisms and are associated with transcriptionally active genes (Henikoff and Shilatifard, 2011). How do these H3K27me3 marks induce silencing of genes expression? The H3K27me3 marks are mainly mediated by Polycomb group (PcG) proteins which cause gene expression by histone modification and nucleosome condensation. Recent studies reported that PcG-mediated H3K27me3 can spread on chromosome and lead to chromatin compaction (Xu et al., 2018). In the absence of PcG genes, the maintenance of chromatin integrity with gene repression by directly associating with target gene loci became lesser extent. The chromatin cannot be a tightly folded structure with lower levels of H3K27me3 modifications (Becker and Workman, 2013; Kingston and Tamkun, 2014). Therefore, more repressive histone marks on a given gene lead to lower transcript levels, whereas less marks cause higher expression levels, which is consistent with our results in this study. Despite K27 or *de novo*_K27 genes, the decreasing in H3K27me3 levels accompanies the de-regulation of gene expression in salt stress treatment (**Figures 3** and **5**). However, *de novo* enrichment of H3K27me3 on target genes leads to repression of expression (**Figures 3** and **5**). These results indicated that H3K27me3 play vital roles in maintaining the appropriate chromatin conformation and integrity, thereby avoiding uncontrolled transcriptional activity when response to abiotic stresses (**Figure 8**).

Indeed, H3K27me3 was negatively correlated with transcript levels in all organisms. A high level of histone H3K27 methylation results in low transcript levels and low H3K27me3 modification levels, lead to actively transcribed genes (Pu and Sung, 2015). In our study, we noticed that most H3K27me3 marked genes were identified in control plants with the basal expression levels (**Supplementary Table 3**). The decrease in repressive H3K27me3 marks of most H3K27me3 genes identified in control plants with up-regulated expression in salt stress is consistent with the notion that the absence of repressive chromatin marks could result in the activation of transcript (**Table 2**). However, the fact that the whole H3K27me3 pattern in salt stress plants was greater than that in control plants was unexpected, although consistent with the differential gene expression pattern (**Figure 4**). In other words, most K27me3 marked genes were mainly those with low expression levels, under stressed conditions. In contrast, genome-wide H3K27me3 pattern in salt treated plants did not show such a trend, indicating that new or *de novo* H3K27me3 marks occurred after salt treatment which may underlie the association of salt-responsive patterns, down-regulation, with differential expression levels. Indeed, we found 650 H3K27me3 marked genes which were not trimethylated in control plants gained H3K27me3 marks after salt stress treatment. The *de novo* methylation has been reported that the new modification can occur in a locus-specific manner during development in yeast, plant and animals (Ooi et al., 2007; Bouyer et al., 2011; Morselli et al., 2015; Stewart et al., 2015). This *de novo* methylation event observed in our study largely shapes the methylation pattern of K27 genes after salt treatment, with additional changes occurring in gene expression required for stress response.

In plants and animals, the histone modification of H3K27me3 maintains the developmentally regulated genes in silenced chromatin status. The removal and establishment of H3K27me3 marks at specific target genes is a dynamic and reversible process and is therefore critically important for normal development. These modifications are carried out by the histone modifiers — histone methyltransferases and histone demethylases. To address how methylation and demethylation were well established in salt stress, we identified some modifiers which may cause changes of H3K27me3 pattern and gene expression observed in our study (**Tables 4** and **5**). In plants, all the HMTs have a well-known conserved SET domain and also named as SET domain groups (SDG) proteins (Thorstensen et al., 2011). In *Arabidopsis*, the methyltransferases trimethylate H3K27, including CLF, MEDEA (MEA) and SWINGER (SWN) (Hennig and Derkacheva, 2009; Liu et al., 2010; Butenko and Ohad, 2011; Zheng and Chen, 2011). Here, we found 43 potential HMT genes with expression in control plants which correspond with different *Arabidopsis* HMTs, such as *CLF*, *ATX*, *ATXR*, *SDG* and *SUVH* (**Table 4**). There are 11 genes which showed different expression pattern in salt stress (**Table 4** and **Figure 6A**). Histone methylation was reversible through the JmjC Jumonji C domain containing proteins and the lysine-specific demethylase (LSD1). Interestingly, we found 21 JmjC genes were expressed in control plants and 4 of them showed differential expression levels (**Table 5** and **Figure 6B**). Among them, *Glyma.04G192000* and *Glyma.20G181000* are homologues of *Arabidopsis* *REF6* and *ELF6* respectively, which are well characterized demethylases. It has been reported that the REF6 protein, also known as JMJ12, can specifically demethylate H3K27me3 at its target gene loci to active gene expression (Lu et al., 2011). The *REF6* mutants cause the ectopic accumulation of H3K27me3 at hundreds of genes and a number of defective developmental phenotypes (Yu et al., 2008; Cui et al., 2016; Li et al., 2016). ELF6 identified as an H3K27me2/3-specific demethylase closely related to *REF6* was rquired for removal of H3K27me3 from the *Flowering Locus C* (*FLC*) locus in developing embryos in vernalized plants (Crevillen et al., 2014). These results suggested that these potential HMT or JmjC protein may function as similar roles to response for establishment or removal of H3K27me3 with conserved mechanisms of the dynamic regulation of H3K27me3 between *Arabidopsis* and soybean.

Based on our results and previous studies, we proposed a hypothesis to illustrate epigenetic regulation of salt stress response in soybean (**Figure 8**). The DNA sequence of genes with low expression levels may be tightly wrapped around the nucleosome and blocked from transcript activation by an unknown mechanism. When plants are subjected to salt stress, for K27 marked genes, decreased levels of H3K27me3 mediated by JmjC proteins release the DNA sequence from the nucleosome for the induced transcription process. DNA sequences of genes with high expression levels are often maintained with a low density of nucleosomes and low levels of inactive histone modification. In contrast, for *de novo*_K27 genes under stress conditions, increased modification levels of inactive marks mediated by HMT proteins on target genes can cause chromatin compaction, thus reducing the gene expression level. However, many details, such as how these JmjC and HMT genes find the proper context and being recruited to establish repressive modification in this hypothesis, need to be clarified by further experiments.

Taken together, our findings described here support a model in which H3K27me3 was closely associated to salt responsive genes under stress conditions in soybean. H3K27me3 modification levels were negatively correlated with the expression level changes of a portion of the salt-responsive genes in soybean. The salt stress can cause *de novo* methylation events in gene regulation for stress response. We identified histone methyltransferases and JmjC domain-containing demethylases in soybean, providing an overview of H3K27me3-mediated salt responsive network. These results suggest that histone modifications may play important but largely unknown roles in the stress responses. It will be of interest to determine and explore how these proteins play roles at specific target genes to mediate local histone methylation enrichment when responding to abiotic stress. The information gathered here will be of particular interest for future studies on the evolution of epigenetic-mediated stress mechanism and the divergence of functionality in crop plants. In addition to the potential roles of histone modifications in influencing stress response, the combination of technical innovation such as synthetic biology and genome editing, will allow greater control of conferring stress tolerance for crop improvement in future agriculture.

## Data Availability

The RNA-seq and histone modification ChIP-seq data sets from this article have been deposited in Gene Expression Omnibus (GEO) with accession number GSE133575.

## Author Contributions

LP and YD conceived and designed the experiments. SL, SG, WW, HZ, TG, QL, XY and FX performed the experiments. LP, LS, GS and WG analyzed data. LP, LS and GS wrote the paper. All authors read and approved the manuscript.

## Funding

This work was supported by National Transgenic Major Program (2019ZX08010-002), the National Key Research and Development Program of China (2016YFD0100103), the Agricultural Science and Technology Innovation Project (CXGC2017JQ018, CXGC2017ZD014), National Natural Science Foundation of China (31872805), National key research and development program (2016YFD0100201-19), Central Public-interest Scientific Institution Basal Research Fund (Y2017JC19) and the Innovation Program of Chinese Academy of Agricultural Sciences.

## Conflict of Interest Statement

The authors declare that the research was conducted in the absence of any commercial or financial relationships that could be construed as a potential conflict of interest.
