# Supplementary material for: Dynamic Changes in Genome-Wide Histone3 Lysine27 Trimethylation and Gene Expression of Soybean Roots in Response to Salt Stress
Source: Front Plant Sci. 2019 Sep 10;10:1031. doi: 10.3389/fpls.2019.01031 (PMC6746917; doi:10.3389/fpls.2019.01031)
Supplement: Supplementary file 4 [file Datasheet_1.pdf]

**Supplementary data**

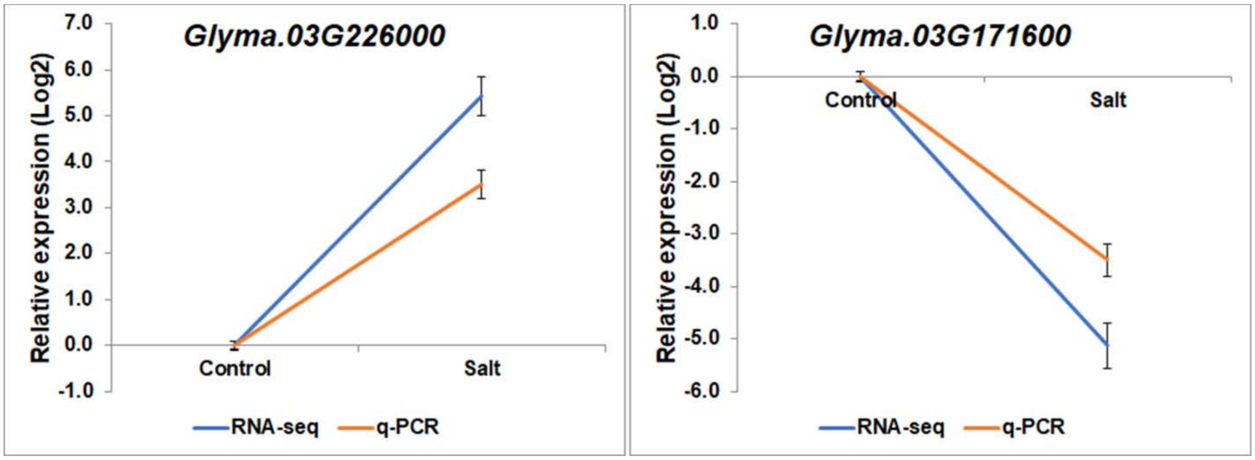


**Supplementary Figure 1.** The gene expression profile of two known soybean salt stress genes analyzed by RNA-seq and q-PCR. Graphs show the relative expression levels of two known soiybean salt genes, (*Glyma. 03G226000* and *Glyma. 03G171600*), analyzed by RNA-seq and by qPCR which normalized to a *Tubulin* (*Glyma.05G203800*) reference gene. Error bars represent standard deviation (SD).

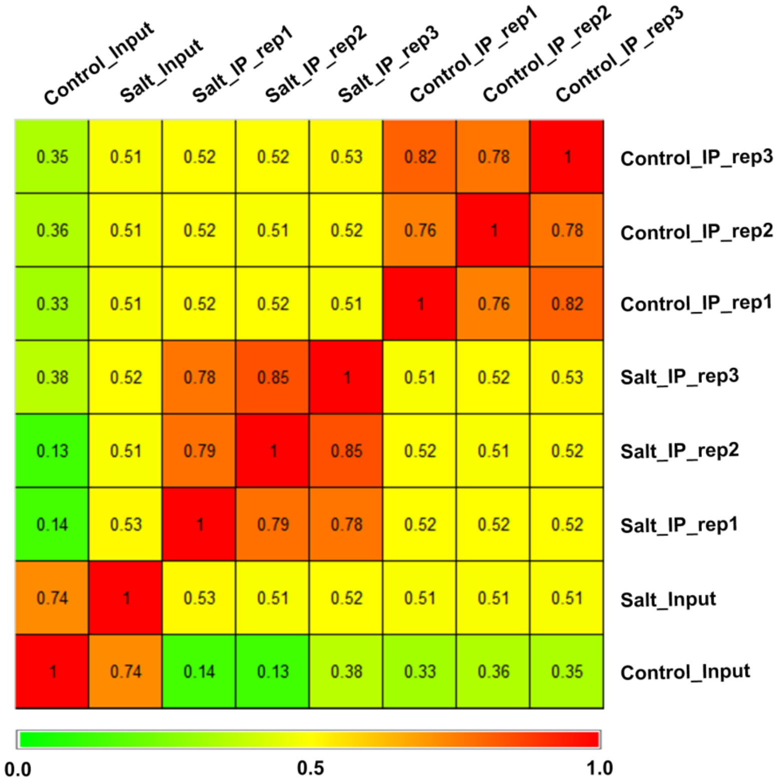


**Supplementary Figure 2.** The correlation coefficient of biological replicates of ChIP-seq data. Verification of ChIP-seq results using Pearson correlation analysis showed high correlation coefficients between the biological replicates for each sample in soybean.

A. Peak distribution

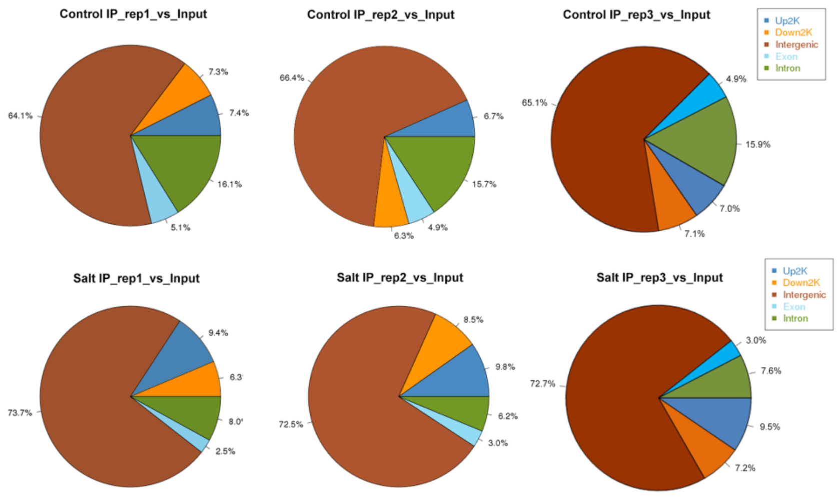


B. Data summary

| Samples | Peak # | Average number | Total length | Average length | Total depth | Annotated gene covered with peaks | Overlapped genes |
|---|---|---|---|---|---|---|---|
| Control | IP_rep1_vs_Input | 2574 | 1807045 | 702.04 | 24344548 | 1707 | 2357 |
| | IP_rep2_vs_Input | 3410 | 2597346 | 761.69 | 49161117 | | |
| | IP_rep3_vs_Input | 2986 | 2197367 | 735.89 | 37865539 | | |
| Salt | IP_rep1_vs_Input | 1262 | 901367 | 714.24 | 15439126 | 746 | |
| | IP_rep2_vs_Input | 1635 | 1148771 | 702.61 | 17422797 | | |
| | IP_rep3_vs_Input | 1458 | 1036404 | 710.84 | 16578646 | | |

**Supplementary Figure 3.** ChIP-seq data characteristics. **(A)** Peak distribution of ChIP-seq analysis with the following genomic regions: promoter (Up2K, 2 kb upstream of the TSS), downstream (Down2K, 2 kb downstream of the TES)， intergenic, exon and intron regions. **(B)** Summary of ChIP-seq data. The number of total genes with H3K27me3 marks in control and/or salt plants is 2357.

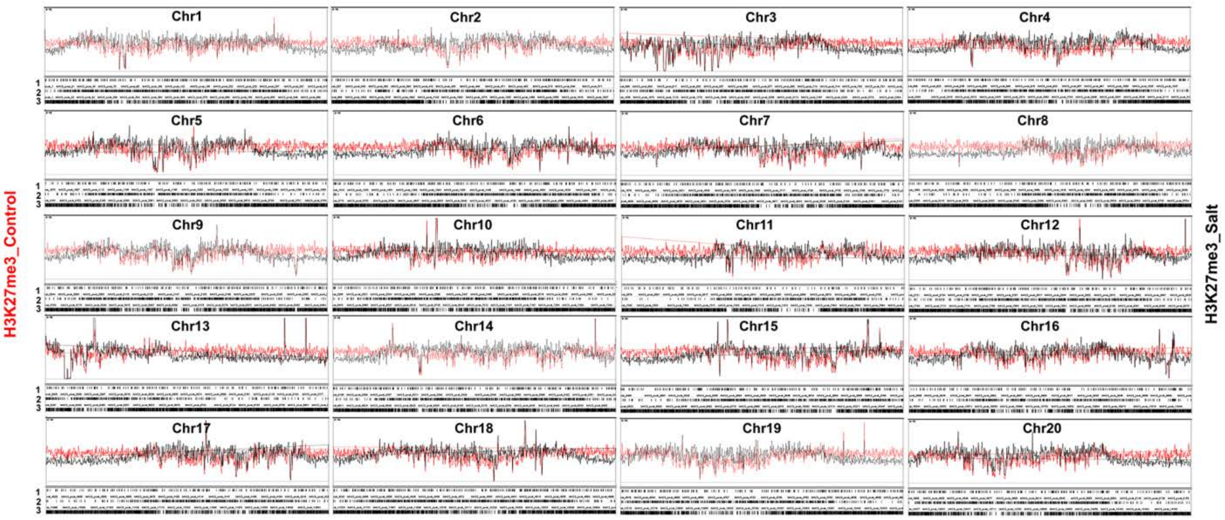


**Supplementary Figure 4.** Chromosomal distribution of H3K27me3 modification sites on all 20 soybean chromosomes. Y-axis represents the input signals for the immunoprecipitation of H3K27me3 in control on the left side (H3K27me3_Control) and salt-treated soybean on the right side (H3K27me3_Salt). The comparison of H3K27me3 marked in control (red) and salt (black) plants were shown on all chromosomes. Chr and 5mb represent chromosome and 5 megabase, respectively. 1 and 2 indicate MACS peaks identified for control (1) and salt (2) samples using MACS with the default $10^{-5}$ p-value cutoff with each input as a control for significant peak calling.

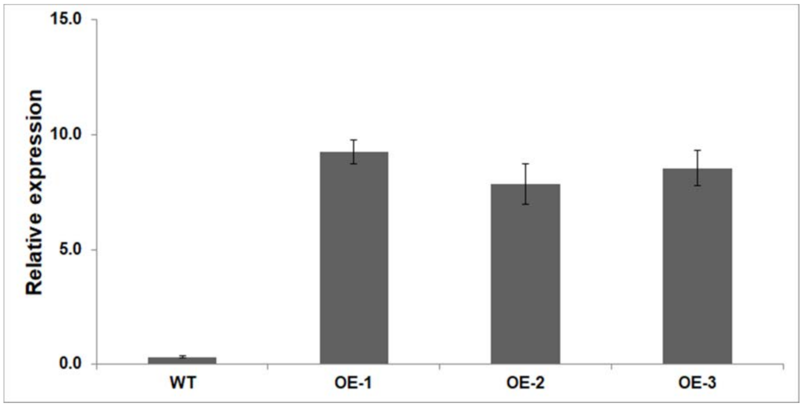


**Supplementary Figure 5.** Expression analysis of *Glyma.17g022500* in transgenic *Arabidopsis*. mRNA expression levels of *Glyma.17g022500* in WT, transgenic *Arabidopsis OE-1, OE-2, OE-3* plants. Graphs show the relative expression levels measured by qPCR, normalized to a *UBIQUITIN* (*UBQ*) reference gene. Error bars represent standard deviation (SD).

**Supplementary Table 1.** List of primer sequences used in the experiments.

| Gene | Sequence | | Purpose |
|---|---|---|---|
| | Forward | Reverse | |
| Glyma.01G188000 | CGCGAATAAAGTGGAGGGTTCA | GCAGTTCCTTCTCTCCGTTGACAT | For qPCR |
| Glyma.01G204900 | GTGGGTTGTGGTTCAACTAATGGT | CCACTGTCAGTTGTTTGACCAAGA | For qPCR |
| Glyma.04G131800 | CCAGAAACCCTACATCTTCGACAT | GGACCTTTTCATCGTATTCGAGG | For qPCR |
| Glyma.04G187000 | TGGCGAGAGGGGAGAGAGC | GCGGTGAGGCTTCATCGG | For qPCR |
| Glyma.04G192000 | CGAGGTTTTGGCATGGCTGAAA | AAGGTGGGAAGGGAGGGATGATTT | For qPCR |
| Glyma.05G203800 | ATGGCTTCGAGCATCCAACA | TGACAGAGGTGCCGCATTTT | For qPCR and ChIP-qPCR |
| Glyma.07G110300 | GAATCAGCGGCAAGAACAACA | GAAGGGTTTGGTGGCAATGTT | For qPCR and ChIP-qPCR |
| Glyma.08G070700 | AGCTTATGATGGCTCACAACAAGG | CACAAATTCTCCCATGTCCCTGT | For qPCR |
| Glyma.08G127000 | GTGAATGAGGAGATAGGGATTGGG | CCACCTCCTTGAACCGAACAAT | For qPCR |
| Glyma.09G041000 | ATTGCTCTTGCTGGTGGCATT | CTGGAAAGCAATGGTGTTGAGTG | For qPCR and ChIP-qPCR |
| Glyma.10G029800 | CCGAAGATATGAAGAGCTTCCAAC | CGGGTGCAGATTGAGATACCATA | For qPCR |
| Glyma.11G204800 | ACCTTGTTGATGTTGTGGTCATGG | GGTCTGCCAAACTTGCGTTTAGAT | For qPCR |
| Glyma.12G104800 | GAGAACCCCATCTTGTGACAAAAG | CCCTCTTTTGACATTTGGCCTT | For qPCR |
| Glyma.13G043800 | CCCTTCTCTCATCTAATTCTGCTGC | CAACTCAAGCACGTACCCTTCTCTT | For qPCR |
| Glyma.14G176700 | TTCTGGAAATTTTGCTGTGGCC | GGGGTGTTTCAATGATCTGTGGTT | For qPCR |
| Glyma.14G213600 | ATGGCGAAAAGGAGCATGTGT | TTATAAGCCGAGTACGTCGCTGA | For qPCR |
| Glyma.19G124100 | TGGAACACCATTTTGGTCAAGG | AGAAACACACACAAAAGGGGCA | For qPCR |
| Glyma.20G168900 | CCAATACAACCAGTTGCTCAGATG | ACATGCATTGTCAGGGTCATCA | For qPCR |
| Glyma.20G181000 | GGTAGTGTTGAAATCCCCAATTGG | GGTTTGGGCAACGGTGGTATAAT | For qPCR |
| Glyma.20G235300 | GGTGCAAAAGGTCCGATGGTAA | ACGACAGTGAATGCGACTTCCTCT | For qPCR |
| Glyma.04G131800 | TTAAGTGCGAGTCCTAACTTCGAGG | AAGCTGCATCAGCCGCATTT | For ChIP-qPCR |
| Glyma.04G187000 | AGTGGTCTTTAAGCTCCAG | GAGTTTGAGGATGGTGAAC | For ChIP-qPCR |
| Glyma.13G043800 | GGTTAATTAGCTCACCTCAGAG | CCAAGCCCTTGAGTCTTAA | For ChIP-qPCR |
| Glyma.14G213600 | ACTATGGTTCGTTGCTTTTGCTTTG | TGCAACACACGAACCACAATTACA | For ChIP-qPCR |
| Glyma.17g022500 | ATGACCTCTGATCATGCTTCCG | AGACCAATGAGAAGGGCAGTGA | For ChIP-qPCR and qPCR |
| | ATGGCTGTGGAAGCATTGTA | TTAAGTGCAATGCCACTG | For full length cDNA |
| UBQ | ATGCAGATTTTCGTGAAAACGC | CAAAGTCGACTCTTTCTGGATG | For qPCR |

**Supplementary Table 2.** Summary of RNA-seq data.

| Sample | Total reads | Quality filtered reads | Uniquely mapped reads | Mappped rate (%) | Correlation coefficient (R) |
|---|---|---|---|---|---|
| Control_rep1 | 46226862 | 44201228 | 42720846 | 96.65 | 1&2=0.85<br>1&3=0.89<br>2&3=0.86 |
| Control_rep2 | 51265272 | 49134852 | 47431628 | 96.53 | |
| Control_rep3 | 50384960 | 48234122 | 46590396 | 96.59 | |

| | | | | | |
|---|---|---|---|---|---|
| Salt_rep1 | 42673942 | 40656396 | 36244302 | 89.15 | 1&2=0.79<br>1&3=0.83<br>2&3=0.85 |
| Salt_rep2 | 54498130 | 50711174 | 48159942 | 94.97 | |
| Salt_rep3 | 51367822 | 48368815 | 44527591 | 92.06 | |

**Supplementary Table 3.** Expression profile of control and salt-treated soybean analyzed by RNA-seq (See excel file).

**Supplementary Table 4.** Summary of ChIP-seq data.

| Sample Name | | Total Reads | Mapped Reads | Mapped Rate (%) | UniqReads |
|---|---|---|---|---|---|
| Control | IP_rep1 | 32309878 | 24359067 | 75.39 | 11323797 |
| | IP_rep2 | 52498286 | 39650520 | 75.53 | 18464272 |
| | IP_rep3 | 53376582 | 41014566 | 76.84 | 19082956 |
| Salt | IP_rep1 | 50123780 | 39909225 | 79.62 | 16346722 |
| | IP_rep2 | 50123476 | 39762243 | 79.33 | 16478284 |
| | IP_rep3 | 51702528 | 41692918 | 80.64 | 17177857 |

**Supplementary Table 5.** The list of genes with H3K27me3 modification in control soybean plants (See excel file).

**Supplementary Table 6.** The list of genes with *de novo* H3K27me3 modification in salt-treated soybean plants (See excel file).
